# Editorial of Special Issue “Sirtuins in Health and Disease”

**DOI:** 10.3390/ijms22105054

**Published:** 2021-05-11

**Authors:** Simon Sedej, Heiko Bugger

**Affiliations:** 1 Department of Cardiology, Medical University of Graz, 8036 Graz, Austria; 2 BioTechMed Graz, 8010 Graz, Austria; 3 Faculty of Medicine, Institute of Physiology, University of Maribor, 2000 Maribor, Slovenia; 4 Faculty of Medicine, University of Freiburg, 79106 Freiburg, Germany

The discovery and characterization of sirtuins as NAD^+^-dependent deacylases have transformed our understanding of post-translational protein regulation. In fact, numerous post-translational modifications have been attributed to the activity of different sirtuins with distinct cellular localization, and recent findings have advanced our knowledge of the importance of sirtuin-catalyzed post-translational modifications in health and disease. Sirtuins have been identified as major regulators of a variety of fundamental intracellular pathways and, thus, play a critical role in aging, energy metabolism, redox homeostasis, inflammation, and in the adaptation to cellular stressors. In this regard, sirtuins have been implicated in the pathology of age-related maladies, including (but not limited to) cancer, diabetes, cardiovascular pathologies, and renal or liver diseases. Based on promising experimental studies, clinical trials have been initiated to (i) assess the potential value of sirtuins as non-invasive biomarkers and (ii) evaluate the therapeutic potential of sirtuin activation.

In this Special Issue, we published an experimental study and a compendium of five review articles covering different aspects for the role of sirtuins in diseases and novel therapeutic strategies.

In this collection, Hong and colleagues comprehensively present experimental studies that have advanced our understanding about the role of sirtuins in the pathogenesis of various kidney diseases, thereby discussing potential molecular mechanisms underlying acute kidney injury, diabetic kidney disease, and renal fibrosis. Current preclinical evidence indicates that sirtuins have a great potential as novel therapeutic targets for the prevention and treatment of age-associated renal diseases [1].

Although sirtuins are increasingly recognized as important players in renal damage driven by hypertension and diabetes, their value as biomarkers has been poorly assessed thus far. In an original article, Martinez-Arroyo and Ortega et al. [2] demonstrated decreased expression of sirtuin1 (SIRT1) in urine sediment from hypertensive patients with urinary albumin excretion, both in patients with or without type 2 diabetes. Interestingly, suppression of SIRT1 expression was also observed in human podocyte cultures subjected to high glucose concentrations and increasing concentrations of angiotensin II. Importantly, decreased levels of SIRT1 were found to be inversely correlated to urinary albumin excretion levels, suggesting that urinary SIRT1 mRNA measurements may reflect incipient renal damage in hypertensive patients. Given the non-invasive nature and thus clinical feasibility of SIRT1 measurements in urinary sediment, decreased urinary SIRT1 levels may have potential value for early detection of renal damage in patients at high risk of developing chronic kidney disease. These findings may stimulate follow-up studies to evaluate a correlation of decreased urinary SIRT1 levels with renal outcomes. This could allow a more detailed appreciation of the prognostic value of urinary SIRT1 both for the development and progression of chronic kidney disease in subjects at high risk.

Besides kidney disease, a growing body of evidence suggests that the antagonistic interplay between SIRT1 and transcription factor NF-κB, a master regulator of inflammatory signaling, may be a promising therapeutic target to control inflammation in hepatic pathologies. De Gregorio et al. [3] concentrate in their review on the modulation of SIRT1 and NF-κB signaling pathways, describing diverse upstream regulators and some natural/synthetic activators of SIRT1 as a possible therapeutic strategy to improve different metabolic and/or inflammatory pathologies in general, and liver diseases in particular.

Emerging evidence indicates that sirtuins, which are predominantly localized within mitochondria, act in synergistic or antagonistic manners to promote respiratory function, antioxidant defense, insulin response, and adipogenesis, and may thereby protect individuals from aging and related metabolic disorders. In this inclusive review, Wang and Wei delineate the mechanisms underlying SIRT3-, SIRT4-, and SIRT5-mediated regulation of mitochondrial function and metabolism, and also discuss the implication of their deficiency in the pathogenesis of insulin resistance and type 2 diabetes [4]. The topic of this article is timely and may stimulate more research exploring the modulation of sirtuins as a preventive or even therapeutic strategy against age-related diseases, such as diabetes mellitus and its complications.

Sirtuins are also involved in carcinogenesis and maintenance of a malignant phenotype, predominantly by regulation of cancer cell viability, apoptosis, tumor metastasis, and tumorigenesis. Despite a high degree of homology among different sirtuins, they may exhibit distinct roles in the various types of cancer. In their current review, De Céu Teixeira et al. [5] focus on the role of SIRT6 in cancer development as one of the sirtuins that has emerged as an important regulator of life expectancy. While SIRT6 has been demonstrated to exert multiple biochemical functions, which interfere with tumorigenesis, the authors highlight the existing evidence that SIRT6 activities may also be engaged in cancer prevention and used for site-specific treatment. Albeit seemingly disparate, this knowledge is essential for elucidating both the potential therapeutic benefits and the detrimental side effects of SIRT6 activation or inactivation. In the second part of their review, the authors discuss recent evidence of the role of SIRT6 in the development of various tumors, thereby underlining the potential use of SIRT6 modulators in cancer nanomedicine. In this regard, one of the outstanding questions is which cancer type requires SIRT6 activation or repression to affect tumor growth.

In aggregate, this Special Issue contains a refreshing combination of articles that present novel data on sirtuins as a biomarker, and discuss current knowledge, existing controversies, and new concepts related to the role of sirtuins in disease pathology and treatment of age-related diseases.
